# Alcohol Drinking and Low Nutritional Value Food Eating Behavior of Sports Bettors in Gambling Advertisements

**DOI:** 10.1007/s11469-017-9789-0

**Published:** 2017-07-12

**Authors:** Hibai Lopez-Gonzalez, Ana Estévez, Susana Jiménez-Murcia, Mark D Griffiths

**Affiliations:** 1 International Gaming Research Unit, Psychology Division, Nottingham Trent University, 50 Shakespeare Street, Nottingham, NG1 4FQ UK; 2 Psychology Department, University of Deusto, Bilbao, Spain; 3 Department of Psychiatry, University Hospital of Bellvitge-IDIBELL, Barcelona, Spain; 4 CIBEROBN, Barcelona, Spain

**Keywords:** Gambling, Sports betting, Marketing, Advertising, Eating, Alcohol

## Abstract

The prevalence of sports betting advertising has become a major concern for gambling regulators, particularly since the legalization of online gambling in many European jurisdictions. Although the composition of gambling advertisement narratives has received some limited attention, nothing is known regarding how betting advertisements (often referred to as “adverts” or “commercials”) might be associating gambling with other potentially risky behaviors. The present paper examines the representation of alcohol drinking and low nutritional value food eating in sports betting advertising. By means of a mixed-methods approach to content analysis, a sample of British and Spanish soccer betting adverts was analyzed (*N* = 135). The results suggest that betting advertising aligns drinking alcohol with sports culture and significantly associates emotionally charged sporting situations such as watching live games or celebrating goals with alcohol. Additionally, alcohol drinking is more frequent in betting adverts with a higher number of characters, linking friendship bonding and alcohol drinking (especially beer) in the context of sports gambling.

Problem gambling is a risky behavior that when engaged in with problem drinking and eating disorders can potentially multiply its detrimental effects on individuals. The associations between the three behaviors remain unclear, although some studies, predominantly among university students, have theorized possible commonalties. For instance, a study conducted with a sample of 246 psychology students found that urgency (no specification whether negative or positive) is the impulsivity-related construct that best predicts alcohol drinking, gambling, and binge eating behavior (Fischer and Smith 2008). Another study with 301 university students showed that problematic gambling and drinking behaviors among students had great similarities, but failed to find any significant underlying cause with eating problems (Hodgins et al. 2016). Also within a higher education context, another study reported that problem student gamblers were more likely to be heavy drinkers than non-problem gamblers (Engwall et al. 2004), an association also identified in nationally representative samples among adolescents in the USA (Barnes et al. 2009). In fact, there is a large literature on the relationship between alcohol and gambling (see Bohaine et al. [2015] for a review) but not in relation to their association in advertisements (also referred to as “adverts” or “commercials”).

At a pathological level, problem gambling is more common among individuals with alcohol use disorder (Grant et al. 2002). Gambling and eating disorders rarely occur simultaneously, although lifetime prevalence of gambling disorder is slightly higher among people with eating disorder (Jiménez-Murcia et al. 2013). However, among individuals with eating disorders underlined by impulse control disorders, such as those with bulimia nervosa, co-morbidity rates with pathological gambling are higher, ranging from 16 to 23% (Jiménez-Murcia et al. 2013), suggesting that purging/binge type of eating and gambling problems might have common environmental and biological risk factors (Claes et al. 2012).

Moving away from the biopsychological underpinnings of gambling interaction with alcohol drinking and eating, other factors also contribute to the association of gambling with similarly risky behaviors. Situational factors (Griffiths 2005) help explain why different environmental settings can affect differently otherwise similar individuals. For instance, comparative studies in Finland and France have shown that the symbolic understanding of problem gambling, alcohol drinking, and eating varies, influencing the way social workers in each country conceptualize each behavior and seek for a solution to those problems (Egerer 2013). One of the most prominent situational factors is arguably advertising and marketing.

The present paper examines sports betting adverts to explore the association of gambling behavior with alcohol drinking and low nutritional value food (LNVF) eating (i.e., eating “junk food”). The study complements the paradigm of individually motivated risky behavior by focusing on the effects that media representation of behavior—by means of repeated patterns of characters, situations, and/or actions in adverts—might have on the imitation and normalization of gambling behavior. Here, the underlying assumption is that sports betting advertising depicts specific gambling behaviors, sometimes in conjunction with LNVF eating and alcohol drinking, creating a symbolic understanding of what gambling on sports means (Lopez-Gonzalez and Griffiths 2016b). This is especially relevant for new products, such as online sports betting, which over the last few years has been legalized in many European jurisdictions (European Commission 2014). New products entail new behavioral patterns that advertisers hope consumers will adopt. Furthermore, advertising plays a major role in communicating socially acceptable ways of consuming such products, as has been noted in prior alcohol and food advertising research (Castonguay et al. 2013; Engels et al. 2009; Mathios et al. 1998).

The available evidence on the interaction of alcohol drinking and LNVF eating in gambling advertising is scarce. Maher and colleagues have argued that sport sponsorship has become a prominent marketing tool for alcohol, gambling, and LNVF. The playing of sport conveys positive healthy connotations to products that pose risks to health (Maher et al. 2006). By specific products (such as alcohol-based drinks, LNVF, and gambling games) associating their brand with sports, companies obscure the risk components of their products, aligning them with the beneficial attributes of physical exercise (McDaniel and Heald 2000). Additionally, gambling venues have been categorized as meeting places where gamblers eat and drink to celebrate their team’s losses and wins (Dyall et al. 2009).

A major concern regarding sports-related marketing of gambling, alcohol, and LNVF is the attraction of children to sport content (Lopez-Gonzalez et al. 2017; Kelly et al. 2011a). Although minors are typically protected from viewing gambling adverts (such as screening them on television only after the 9pm), many jurisdictions specifically permit betting promotions during sport events, even if these events are broadcast before 9 pm (Lopez-Gonzalez and Griffiths 2016a). Research investigating three televised sporting events in Australia in 2012 found that spectators were exposed to gambling, alcohol, or LNVF products for about two thirds of the overall broadcast time, resulting in 4062 episodes of alcohol marketing, 322 for gambling, and 51 for LNVF, including different marketing forms such as match commentary, banners, shirt sponsors, adverts, and on-screen messages (Lindsay et al. 2013). Interviews with children aged 10–14 years have shown that they are more likely to recall food than non-food sponsors of sport teams, while children aged 10–11 years thought it was important to buy food or beverages from sport sponsors to “return the favor” (Kelly et al. 2011b). In experiments with 85 children aged 5–12 years, one study found that the most liked teams were associated in children’s minds to junk food products, and that continued exposure to gambling, and that food and drink adverts fostered connections between consuming those products and sport (Bestman et al. 2015).

Although some sport codes prohibit the depiction of alcohol drinking in adverts, past research has demonstrated that fans can associate drinking with sport even when characters in the adverts are not shown physically drinking, due to the depiction of scenarios or situations that evoke alcohol drinking in their minds (Zwarun and Farrar 2005). Also, drinking alcohol is usually represented as being associated with friendship and camaraderie (Zwarun and Farrar 2005), an association that mirrors that of gambling adverts (Deans et al. 2016; Hing et al. 2017; Sproston et al. 2015).

Building on these findings, two exploratory research questions (RQs) were proposed in the present paper to understand how sports betting advertising represents bettors’ behavior. RQ1: How is eating LNVF and drinking alcohol in sports betting adverts represented in sport culture. In particular, this question seeks to explore whether sports betting adverts present gambling, eating LNVQ, and drinking alcohol in association with emotionally charged situations, such as watching live sport. RQ2: How is drinking alcohol and eating LNVF portrayed in sports betting adverts in relation to friendship building.

## Method

### Sample

Sports betting television adverts (*N* = 135) were sampled from a larger cross-sectional study. Advertisements with an upload date from June 2014 to November 2016 were downloaded from the official *YouTube* channels of 29 different betting brands. These brands were primarily selected based on their presence as sponsors, official partners, or regular advertisers in sport events. All adverts met the following inclusion criteria: (i) only those advertising soccer were included, thus excluding horse and dog racing as well as other sports whose popularity is more country-specific; (ii) only advertisements from Spain and the UK were selected because these were the two languages that the authors could understand as native speakers, and the representativeness of La Liga and Premier League competitions in European soccer (first and third, respectively, in Union of European Football Associations [UEFA] ranking); (iii) the advertisements belonged to companies with a legal license to operate in one or both of the countries analyzed; (iv) the adverts had to have a televisual and temporal format (between 20 and 60 s duration). This latter inclusion criterion excluded made-for-internet promotions that typically allow informal shooting or discussion-like videos including tipsters sponsoring a brand. Also, longer advertisements were excluded because they were unlikely to have been shown on television; and (v) for the purpose of assessing new behavioral patterns attached to new products, the advertisements had to focus on online sports betting, excluding those addressing offline betting. Further details of the adverts analyzed can be obtained from the first author on demand.

To check if the *YouTube* sample corresponded to the actual adverts shown on television, an additional sample of nine UEFA Champions League, English Premier League, and Spanish La Liga soccer matches (including pre- and post-match advert breaks) was recorded from May to June 2016. The results confirmed that every television advertisement was made accessible, sometimes with a few weeks’ delay, via the operator’s *YouTube* profile. Also, the electronic banners around the soccer field were analyzed to make sure no betting brand being promoted was excluded from the study sample.

### Procedure

The initial analysis was conducted by three coders. Coder 1 rated the whole sample of advertisements. For reliability purposes, a sub-sample composed 23 adverts (17.03%) was randomly generated and coded independently by coders 2 and 3. Interrater reliability was calculated using ReCal3 software, which is especially designed for situations with three or more coders analyzing nominal data wherein Cronbach’s alpha is not useful. In the present study, a solid interrater reliability was found. Krippendorff’s alpha coefficients ranged from 0.78 to 1, with all except one variable punctuating below the 0.80 cut-off point generally recommended for acceptable reliability, but enough for exploratory analysis (Krippendorff 2013).

Given the exploratory nature of the study, a mixed-methods approach was favored by the researchers for the second round of analysis. Thus, once the betting adverts portraying alcohol and LNVF consumption were identified, coder 1 conducted additional qualitative coding on them to deepen the exploratory questions that guided the initial analysis. The definition of LNVF included snacks (e.g., crisps, popcorn, etc.), sugary drinks, pizza, hamburgers, and French fries.

To assess the association between alcohol and LNVF with sports culture (RQ1), characters in the adverts consuming alcohol or LNVF were hypothesized to be associated with (1) having a sentimental bond with sport (e.g., team loyalty); (2) being depicted as in an emotional state; (3) appearing celebrating a goal; (4) betting while viewing sport (i.e., in-play betting); (5) being offered by the advert free bets, money back, or similar promotions; and (6) showing satisfaction from the outcome of a bet or a game. To explore RQ2, namely the association between unhealthy food and alcohol with friendship building, variables 1 to 6 were hypothesized to vary depending on the number of characters in adverts portrayed drinking alcohol; and similarly on the number of characters in adverts portrayed eating LNVF. Additionally, adverts were analyzed in terms of (7) the type of drink consumed, under the assumption that drinking beer (e.g., lager, cider) will be more likely to occur in group settings in contrast to drinking wine or spirits.

Chi-square tests were performed between the variables outlined above using IBM SPSS 23 for Mac. Fisher’s exact test (one-sided) was used when the distribution expected cell counts were below 5. Considering the exploratory purpose of the study, and given the scarcity of scientific literature in the field and the mixed-methods approach, a threshold *p* value lower than 0.05 was accepted as statistically significant without controlling for multiple comparisons error.

## Results

Initial analysis of the 135 adverts identified 36 betting adverts depicting LNVF consumption (26.7%), and 30 betting adverts depicting alcohol consumption (22.2%). The incidence of simultaneous alcohol drinking and LNVF eating was relatively low, comprising only 11 adverts (8.14%). LNVF eating behavior showed little association with any of the variables analyzed. Sentimental identification with the team, emotionally charged scenes, in-play betting situations, and free bet promotions, had no statistically significant association with LNVF eating behavior. Only two variables were suggestive of an association. More specifically, (i) LNVF eating was associated with the characters in the advert celebrating a goal (*χ* ^2^[2, *N* = 36] = 6.401, *p* < .041); and (ii) the number of characters in the advert eating LNVF was associated with showing satisfaction to what could be implied as a satisfactory game or bet outcome (*χ* ^2^[6, *N* = 36] = 16.107, *p* < .013).

In contrast, alcohol drinking showed several associations with sports betting behavior. The presence of alcohol in the advert co-occurred with (i) the appearance of characters with a sentimental bond (*χ* ^2^[1, *N* = 135] = 5.310, *p* < .026); (ii) the characters in the advert watching live sport while betting (i.e., in-play betting) (*χ* ^2^[1, *N* = 135] = 6.199, *p* < .011); (iii) any of the characters celebrating a goal (i.e., only fans, excluding those characters playing the part of a footballer) (*χ* ^2^[1, *N* = 135] = 3.978, *p* < .042); and (iv) the character showing satisfaction for what appears to be the outcome of a bet or a game (*χ* ^2^[2, *N* = 135] = 6.603, *p* < .033). The provision of free bets (or other similar promotions) and how emotionally charged the main character appeared to be did not yield any statistically significant relationship with alcohol drinking behavior.

Further analysis showed that a higher number of characters in the adverts that depicted alcohol drinking was associated with (i) the main character being more emotionally involved (*χ* ^2^[3, *N* = 30] = 19.566, *p* < .001); (ii) the characters celebrating a goal (*χ* ^2^[2, *N* = 30] = 6.589, *p* < .037); (iii) the characters watching live sport while betting (*χ* ^2^[3, *N* = 30] = 9.830, *p* < .020); and (iv) the advert offering free bets (*χ* ^2^[3, *N* = 30] = 9.037, *p* < .029); and (5) drinking beer (*χ* ^2^[3, *N* = 30] = 13.083, *p* < .004). The number of characters drinking was not statistically associated with the (i) appearance of characters with a sentimental bond, or (ii) showing of satisfaction due to bet or game outcome.

Alcohol type was further analyzed, binary coding whether the adverts portrayed beer drinking (e.g., lager, cider) as opposed to wine/spirits drinking. Those adverts with characters drinking beer were associated with (i) a higher number of overall characters in the advert (*χ* ^2^[3, *N* = 30] = 13.083, *p* < .004); (ii) free money offers (*χ* ^2^[1, *N* = 30] = 4.751, *p* < .043); and (iii) characters celebrating a goal (*χ* ^2^[1, *N* = 30] = 9.455, *p* < .003). Contrary to what was hypothesized, in-play betting, emotional involvement of the main character, and sentimental identification were not statistically associated with beer drinking. However, among the 11 adverts showing both alcohol and LNVF consumption, drinking beer was not only associated with eating LNVF (*χ* ^2^[1, *N* = 11] = 5.687, *p* < .020), but also with a higher number of characters eating LNVF in the advert (*χ* ^2^[2, *N* = 11] = 11.000, *p* < .004). Among the 11 adverts showing wine/spirits drinking, only one (9.09%) also depicted LNVF consumption, whereas among the 19 adverts with characters drinking beer, 10 also consumed LNVF (52.63%).

## Discussion

The present study provides a preliminary and tentative understanding of how sports betting adverts depict gambling in interaction with eating and drinking behavior. Approximately one-quarter of the analyzed sample depicted alcohol (22.2%) and LNVF (26.7%) consumption. However, when combined, 55 out of 135 adverts (40.7%) depicted at least one of the two behaviors. Gambling, like consuming LNVF and alcohol, is largely considered a risky product whose marketing and advertising must be limited (Hing et al. 2014, 2015, 2016). Characters in gambling narratives, as seen in the adverts sampled, can help to create a representational atmosphere of normality regarding the simultaneous use of two or more of such potentially risky products. Also, the transmission of such adverts does not happen in isolation. On the contrary, they add to existing alcohol and food products, which are also heavily advertised in the context of sport events (Bestman et al. 2015; Lamont et al. 2011; Sproston et al. 2015; Thomas et al. 2012).

Under the assumptions for RQ1, alcohol drinking was hypothesized to be associated with sports culture. The analyses showed that betting adverts portraying alcohol consumption, as opposed to those without alcohol, showed (i) more frequently sentimental bonds between characters and sport; (ii) more frequent betting while watching a live game; (iii) more goal celebrations; and (iv) in general, characters demonstrating greater satisfaction for the outcome of the games or bets. These activities were examined in order to explore the representation of bettors’ behavior in emotionally charged situations. Sport is far from being an emotionally neutral content, and often entails identity and belonging aspects (Giulianotti et al. 2005; Wenner 2011). Thus, betting advertising appears to capitalize on such sentiments arising from sports culture and aligns them with betting culture, corroborating recent research on the field (Deans et al. 2016, 2017). In addition, emotionally charged situations such as betting while viewing a game or celebrating a goal are arguably more likely to be associated with instant decision-making and urgency, potentially harming those with impulse control disorders (Hodgins et al. 2016). Obviously, the advertising and marketing industry is a manufacturer of happy-ending stories, and as such, gambling, eating, and drinking are unlikely to be portrayed in a context of excessive or disordered consumption. However, against a gambling backdrop, the simultaneous representation of emotional situations and alcohol consumption, even moderate, could also encourage disinhibition.

Friendship building was also hypothesized in relation to the number of characters drinking alcohol in the adverts, as proposed in RQ2. Simply put, those adverts that depicted alcohol drinking featured (on average) more characters in their narratives than those adverts with no alcohol drinking. Solitary gambling, similar to solitary alcohol drinking, exposes individuals to greater risks of developing problems (Griffiths 1995). However, studies have raised concerns regarding the misconception that drinking, especially beer, when done socially cannot be harmful (Strate 1992), reminiscent of the bettors’ idea that gambling in group is *safer,* as expressed in previous qualitative research (e.g., Deans et al. 2016). In this context, betting with friends might also add to the security component of offers such as guaranteed money back or free bet coupons, explaining why they are more likely to be offered in such circumstances.

Bonding with peers has been previously been proposed as a major component of gambling advertising, both in sports (Lindsay et al. 2013) as well as in other forms of gambling (McMullan and Miller 2008). Prototypical betting advertising representations continue to portray betting and sport viewing as a communal experience, an image that appears to be more aspirational than realistic considering that the majority of people consume sport alone (ESPN 2010). Group behavior, contrary to the idea implied in betting adverts sampled in the present study, could facilitate the feedback and normalization of a vicious circle of risky behaviors, as suggested by the fact that adverts with more characters drinking also were more likely associated with LNVF eating.

The relatively small sample of adverts analyzed cannot be ignored when acknowledging the limitations of the study. The data here can only be interpreted as a preliminary and tentative examination of the simultaneous presence of gambling, alcohol, and LNVF in social representations that have the capacity to affect bettors’ behavior, particularly those more vulnerable such as problem gamblers and minors. Future research will need to evaluate whether new forms of advertising are having an impact on the co-occurrence of these three behaviors.

## Conclusion

To the authors’ knowledge, this is the first research study that has explored the interaction of drinking alcohol and eating unhealthy food in gambling adverts. It is argued that situational factors such as the advertising and marketing of gambling can help to shape betting behavior, by means of depicting specific scenarios and actions, while ignoring others, that show characters drinking alcohol and betting, and less frequently, eating unhealthy food. It has been further argued that drinking alcohol is typically shown in betting adverts in clear association with sports culture and gambling. Similarly, betting advertising might be resorting to characters drinking alcohol, largely beer, to enhance the message of friendship bonding that appears to be inherently associated with the enjoyment of sport. Media and advertising regulators (as well as policymakers more generally) should be cognizant and react to the symbolic linkage of independent risky behaviors, particularly in contexts of enhanced emotions and impulses, such as popular sports, and considering the appeal of such content to children.

## References

[CR1] Barnes GM, Welte JW, Hoffman JH, Tidwell M-CO (2009). Gambling, alcohol, and other substance use among youth in the United States. Journal of Studies on Alcohol and Drugs.

[CR2] Bestman A, Thomas SL, Randle M, Thomas SDM (2015). Children’s implicit recall of junk food, alcohol and gambling sponsorship in Australian sport. BMC Public Health.

[CR3] Bohaine G, Guerrier Y, Sakhuja R, Vamvakari T (2015). The relationship between alcohol and gambling behaviors.

[CR4] Castonguay J, Kunkel D, Wright P, Duff C (2013). Healthy characters? An investigation of marketing practices in children’s food advertising. Journal of Nutrition Education and Behavior.

[CR5] Claes L, Jimenez-Murcia S, Agüera Z, Sánchez I, Santamaría J, Granero R, Fernández-Aranda F (2012). Eating disorders and pathological gambling in males: can they be differentiated by means of weight history and temperament and character traits?. Eating Disorders.

[CR6] Deans EG, Thomas SL, Daube M, Derevensky J, Gordon R (2016). Creating symbolic cultures of consumption: an analysis of the content of sports wagering advertisements in Australia. BMC Public Health.

[CR7] Deans, E. G., Thomas, S. L., Daube, M., & Derevensky, J. (2016b). The role of peer influences on the normalisation of sports wagering: a qualitative study of Australian men. *Addiction Research & Theory.* doi:10.1080/16066359.2016.1205042.

[CR8] Deans EG, Thomas SL, Derevensky J, Daube M (2017). The influence of marketing on the sports betting attitudes and consumption behaviours of young men: implications for harm reduction and prevention strategies. Harm Reduction Journal.

[CR9] Dyall L, Tse S, Kingi A (2009). Cultural icons and marketing of gambling. International Journal of Mental Health and Addiction.

[CR10] Egerer M (2013). Problem drinking, gambling and eating—three problems, one understanding? A qualitative comparison between French and Finnish social workers. Nordic Studies on Alcohol and Drugs.

[CR11] Engels RCME, Hermans R, van Baaren RB, Hollenstein T, Bot SM (2009). Alcohol portrayal on television affects actual drinking behaviour. Alcohol and Alcoholism.

[CR12] Engwall D, Hunter R, Steinberg M (2004). Gambling and other risk behaviors on university campuses. Journal of American College Health.

[CR13] ESPN (2010). ESPN top ten list for sport research. Integrated media research report.

[CR14] European Commission (2014). Recommendation on principles for the protection of consumers and players of online gambling services and for the prevention of minors from gambling online.

[CR15] Fischer S, Smith GT (2008). Binge eating, problem drinking, and pathological gambling: linking behavior to shared traits and social learning. Personality and Individual Differences.

[CR16] Giulianotti R, Bonney N, Hepworth M (2005). Football, violence and social identity.

[CR17] Grant JE, Kushner MG, Kim SW (2002). Pathological gambling and alcohol use disorder. Alcohol Research and Health.

[CR18] Griffiths MD (1995). Adolescent gambling.

[CR19] Griffiths MD (2005). A biopsychosocial approach to addiction. Psyke & Logos.

[CR20] Hing N, Vitartas P, Lamont M (2014). Promotion of gambling and live betting odds during televised sport: influences on gambling participation and problem gambling.

[CR21] Hing N, Lamont M, Vitartas P, Fink E (2015). Sports bettors’ responses to sports-embedded gambling promotions: implications for compulsive consumption. Journal of Business Research.

[CR22] Hing N, Russell AMT, Vitartas P, Lamont M (2016). Demographic, behavioural and normative risk factors for gambling problems amongst sports bettors. Journal of Gambling Studies.

[CR23] Hing, N., Russell, A. M. T., Lamont, M., & Vitartas, P. (2017). Bet anywhere, anytime: an analysis of internet sports bettors’ responses to gambling promotions during sports broadcasts by problem gambling severity. *Journal of Gambling Studies.* doi:10.1007/s10899-017-9671-9. 10.1007/s10899-017-9671-9 28150058

[CR24] Hodgins DC, von Ranson KM, Montpetit CR (2016). Problem drinking, gambling and eating among undergraduate university students. What are the links?. International Journal of Mental Health and Addiction.

[CR25] Jiménez-Murcia S, Steiger H, Isräel M, Granero R, Prat R, Santamaría JJ (2013). Pathological gambling in eating disorders: prevalence and clinical implications. Comprehensive Psychiatry.

[CR26] Kelly B, Baur LA, Bauman AE, King L, Chapman K, Smith BJ (2011). Food and drink sponsorship of children’s sport in Australia: who pays?. Health Promotion International.

[CR27] Kelly B, Baur LA, Bauman AE, King L, Chapman K, Smith BJ (2011). “Food company sponsors are kind, generous and cool”: (Mis)conceptions of junior sports players. International Journal of Behavioral Nutrition and Physical Activity.

[CR28] Krippendorff K (2013). Content analysis: an introduction to its methodology.

[CR29] Lamont M, Hing N, Gainsbury S (2011). Gambling on sport sponsorship: a conceptual framework for research and regulatory review. Sport Management Review.

[CR30] Lindsay S, Thomas S, Lewis S, Westberg K, Moodie R, Jones S (2013). Eat, drink and gamble: marketing messages about “risky” products in an Australian major sporting series. BMC Public Health.

[CR31] Lopez-Gonzalez H, Griffiths MD (2016). Is European online gambling regulation adequately addressing in-play betting advertising?. Gaming Law Review and Economics.

[CR32] Lopez-Gonzalez, H., & Griffiths, M. D. (2016b). Understanding the convergence of online sports betting markets. *International Review for the Sociology of Sport*. doi:10.1177/1012690216680602.

[CR33] Lopez-Gonzalez H, Estévez A, Griffiths MD (2017). Marketing and advertising online sports betting: a problem gambling perspective. Journal of Sport and Social Issues.

[CR34] Maher, A., Wilson, N., Signal, L., & Thomson, G. (2006). Patterns of sports sponsorship by gambling, alcohol and food companies: an internet survey. *BMC Public Health, 6*. doi:10.1186/1471-2458-6-95. 10.1186/1471-2458-6-95 PMC1459130 16608525

[CR35] Mathios A, Avery R, Bisogni C, Shanahan J (1998). Alcohol portrayal on prime-time television: manifest and latent messages. Journal of Studies on Alcohol.

[CR36] McDaniel SR, Heald GR (2000). Young consumers’ responses to event sponsorship advertisements of unhealthy products: implications of schema-triggered affect theory. Sport Management Review.

[CR37] McMullan JL, Miller D (2008). All in! The commercial advertising of offshore gambling on television. Journal of Gambling Issues.

[CR38] Sproston K, Hanley C, Brook K, Hing N, Gainsbury S (2015). Marketing of sports betting and racing.

[CR39] Strate L, Craig S (1992). Beer commercials: a manual on masculinity. Men masculinity and the media.

[CR40] Thomas S, Lewis S, Duong J, McLeod C (2012). Sports betting marketing during sporting events: a stadium and broadcast census of Australian Football League matches. Australian and New Zealand Journal of Public Health.

[CR41] Wenner LA, Billings A (2011). Mocking the fan for fun and profit. Sports dirt, fanship identity, and commercial narratives. Sports media. Transformation, integration, consumption.

[CR42] Zwarun L, Farrar KM (2005). Doing what they say, saying what they mean: self-regulatory compliance and depictions of drinking in alcohol commercials in televised sports. Mass Communication and Society.

